# Appendicitis

**Published:** 1904-08-13

**Authors:** 


### APPENDICITIS.

Cases of pain in the abdomen are so common, so difficult to discriminate, and frequently so unsatisfactory to treat that Mr. Bowlby's [1] remarks anent vague pains associated with an inflamed appendix will be of very general interest. He speaks of a boy who had occasional attacks of "acute indigestion" for two years. At night or perhaps in the early morning he would have pain in the abdomen and sickness. One attack following measles was more severe and he complained of pain and there was tenderness in his right iliac fossa. His temperature rose to 102·6° F. Next morning he was well again. This boy's appendix was removed and examined, it was very long, contained some fæcal material and was distended near its tip. There was no evidence of peritoneal inflammation. The explanation of the attacks of pain seems to be that the appendix from time to time got temporarily blocked and distended with secretion and fæcal material. One girl, whose appendix showed no sign of inflammation, but was swollen and sharply kinked by a very short mesentery, had four attacks of pain localised on the right side of the abdomen and accompanied by fever, sickness, and constipation. Attacks were probably induced by catarrh of the appendix.

Another class of case is that in which the patient has recovered from a very severe attack of acute appendicitis during the course of which he has passed pus by the bowel, and the question arises:—Is he liable to another attack? Mr. Bowlby states his belief that in such a case recurrence is highly improbable, for the violence of the inflammation usually destroys the appendix entirely, and the pus found in the dejecta shows that an abscess has formed and burst into the bowel. Indeed, if it be found that an adult has had a very violent and prolonged attack of such severity as to threaten his life, the probability is that there may be no necessity to operate for the removal of his appendix. Painful micturition is frequent in cases of acute appendicitis, and indicates that the inflammation has spread into the pelvis; very painful micturition is suggestive of a pelvic abscess, and on examination per rectum may confirm such a diagnosis.

A patient may apparently have got over the worst of his attack of appendicitis, there may be no bad pain, no pyrexia, but if the pulse remains frequent, though not increasing perhaps, and there be rigidity of the abdominal muscles, and especially if the sickness returns when mouth feeding has been discontinued it is much better to operate than to wait. Pus will almost certainly be found in the region of the appendix.

[1] Lancet, July 9, 1904.

